# Neuroprotective Properties of Clove (*Syzygium aromaticum*): State of the Art and Future Pharmaceutical Applications for Alzheimer’s Disease

**DOI:** 10.3390/biom15030452

**Published:** 2025-03-20

**Authors:** Tatevik Sargsyan, Hayarpi M. Simonyan, Lala Stepanyan, Avetis Tsaturyan, Caterina Vicidomini, Raffaele Pastore, Germano Guerra, Giovanni N. Roviello

**Affiliations:** 1 Scientific and Production Center “Armbiotechnology” NAS RA, 14 Gyurjyan Str., Yerevan 0056, Armenia; tatev-sargsyan@ysu.am (T.S.); lala_stepanyan@rambler.ru (L.S.);; 2 Institute of Pharmacy, Yerevan State University, 1 Alex Manoogian Str., Yerevan 0025, Armenia; 3 Institute of Biostructures and Bioimaging, Italian National Council for Research (IBB-CNR), Area di Ricerca Site and Headquarters, Via Pietro Castellino 111, 80131 Naples, Italy; 4 Department of Medicine and Health Sciences “Vincenzo Tiberio”, University of Molise, Via F. De Santis, 86100 Campobasso, Italy

**Keywords:** cloves, herbal medicine, nutrition, Alzheimer’s disease, neurodegeneration

## Abstract

This study explores the neuropharmacological potential of various molecular and amino acid components derived from *Syzygium aromaticum* (clove), an aromatic spice with a long history of culinary and medicinal use. Key bioactive compounds such as eugenol, α-humulene, β-caryophyllene, gallic acid, quercetin, and luteolin demonstrate antioxidant, anti-inflammatory, and neuroprotective properties by scavenging free radicals, modulating calcium channels, and reducing neuroinflammation and oxidative stress. Moreover, gallic acid and asiatic acid may exhibit protective effects, including neuronal apoptosis inhibition, while other useful properties of clove phytocompounds include NF-κB pathway inhibition, membrane stabilization, and suppression of pro-inflammatory pathways, possibly in neurons or other relevant cell types, further contributing to neuroprotection and cognitive enhancement. Amino acid analysis revealed essential and non-essential amino acids such as aspartic acid, serine, glutamic acid, glycine, histidine, and arginine in various clove parts (buds, fruits, branches, and leaves). These amino acids play crucial roles in neurotransmitter synthesis, immune modulation, antioxidant defense, and metabolic regulation. Collectively, these bioactive molecules and amino acids contribute to clove’s antioxidant, anti-inflammatory, neurotrophic, and neurotransmitter-modulating effects, highlighting its potential as a preventive and therapeutic candidate for neurodegenerative disorders. While preliminary preclinical studies support these neuroprotective properties, further research, including clinical trials, is needed to validate the efficacy and safety of clove-based interventions in neuroprotection.

## 1. Introduction

Neurodegenerative diseases are a global health challenge, affecting millions and imposing significant burdens on healthcare systems, families, and economies. They are particularly prevalent in Western societies, where factors like aging populations may contribute to their high incidence [1]. These disorders, including Alzheimer’s disease (AD), Parkinson’s disease (PD), Huntington’s disease (HD), and amyotrophic lateral sclerosis (ALS), progressively deteriorate nerve cells in the brain and spinal cord, leading to cognitive decline, motor impairments, and often fatal consequences. The economic implications of neurodegenerative disorders are substantial, with immense costs associated with patient care, medication, long-term assistance, and loss of productivity [2]. These expenses strain healthcare systems and exert financial pressure on families, significantly affecting their quality of life [3,4,5,6]. While advancements in research and healthcare have significantly improved the diagnosis and management of neurodegenerative diseases, the absence of effective disease-modifying treatments remains a critical challenge. This dichotomy reflects the progress made in understanding the pathology of neurodegenerative diseases and the ongoing difficulties in translating this knowledge into therapies that alter their trajectory. The complex nature of these diseases demands better therapeutic strategies, emphasizing the urgency for innovative interventions, as AD is a progressive neurodegenerative disorder that represents one of the most pressing healthcare challenges of our time. It is believed to be a disorder characterized by the aggregation of amyloid-beta (Aβ) plaques and neurofibrillary tangles, which are associated with synaptic dysfunction, neuronal loss, and cognitive impairment [7]. Genetic factors, such as mutations in the amyloid precursor protein (APP) and presenilin genes, play a role in early-onset AD, while aging, environmental factors, and lifestyle contribute to late-onset AD [8,9]. Current AD therapies face significant limitations. Cholinesterase inhibitors (donepezil, rivastigmine, galantamine) and the N-methyl-D-aspartate (NMDA) receptor antagonist (memantine [10]) are approved drugs for AD symptomatic treatment [1]. However, these medications do not modify the underlying disease pathology. Recently, drugs such as aducanumab and lecanemab have been approved, although their benefits appear to be modest, primarily slowing cognitive decline rather than reversing or halting the progression of Alzheimer’s disease [11,12]. These therapies target amyloid plaques, a hallmark of Alzheimer’s, but their impact on long-term disease progression remains uncertain. Additionally, the most of therapies are often invasive (e.g., intravenous administration) and can have severe side effects, such as brain swelling and bleeding [13]. Symptomatic treatments for memory loss and neuropsychiatric symptoms provide temporary relief, but do not address underlying neurodegeneration. Donanemab, an amyloid β-directed antibody, received approval in the USA for the treatment of adults with early symptomatic Alzheimer’s disease, but the National Institute for Health and Care Excellence has recently not recommended it for use in the National Health Service (NHS) in the United Kingdom due to its relatively small benefits and high rollout costs, including regular infusions and side effect monitoring [14]. Advances in disease-modifying therapies remain critical to improving outcomes [15,16]. In this context, nature-inspired and plant-based therapies [17,18,19,20] are gaining attention for their potential in treating AD, offering a natural and holistic approach to a condition with limited pharmaceutical solutions [21,22]. Addressing socially relevant diseases drives the search for new therapeutic strategies, which are based on molecular systems that include not only natural, but also synthetic compounds, such as peptidic and oligonucleotidic molecules, as well as hybrid structures like nucleopeptides [23,24,25,26,27,28,29,30,31,32,33]. However, plants like Ginkgo biloba, Huperzine A, ginseng, and turmeric contain bioactive compounds that address several key aspects of AD’s pathology, including oxidative stress, neuroinflammation, and Aβ plaque formation [34]. Ginkgo biloba, one of the most studied herbs for AD, is known for its flavonoids and terpenoids that act as powerful antioxidants and anti-inflammatory agents. Clinical trials have shown that extracts like EGb 761 can improve cognitive functions in mild-to-moderate dementia and alleviate neuropsychiatric symptoms, though results vary, highlighting the need for more rigorous studies [35,36]. Similarly, Huperzine A, derived from the Chinese club moss, shows promise as it enhances cholinergic signaling in the brain by inhibiting acetylcholinesterase, though long-term efficacy remains to be fully understood [37]. Ginseng, which is rich in ginsenosides, has demonstrated the ability to reduce Aβ accumulation and oxidative damage both critical in AD progression [38,39]. Meanwhile, curcumin, the active compound in turmeric, has been noted for its anti-inflammatory properties and potential to reduce plaque burden although its poor bioavailability poses a challenge [40,41,42]. Sage (*Salvia officinalis*) is another herb that has shown benefits in improving memory and cognitive performance in clinical trials alone and in addiction with *Hypericum perforatum*, also known as St. John’s Wort [43,44]. The strength of herbal therapies lies in their ability to target multiple pathways involved in AD’s complex mechanisms. Among the various underlying mechanisms of AD, mitochondrial dysfunction plays a crucial role. This includes factors such as increased production of reactive oxygen species (ROS) [45,46], disrupted calcium balance, and disturbances in mitochondrial dynamics [47]. In this context, the clove (*Syzygium aromaticum*) [48] and its primary bioactive compound, eugenol [49], provided very interesting results for their potential therapeutic effects on AD. These effects are primarily linked to clove’s antioxidant and anti-inflammatory properties, which address two critical pathological processes in AD: oxidative stress and neuroinflammation [50]. Eugenol has been shown to neutralize free radicals and reduce inflammatory cytokines, thereby protecting neurons from damage associated with Aβ plaques and tau protein aggregation, hallmarks of AD [51]. Preclinical studies suggest that clove essential oil can attenuate cognitive decline and improve memory functions in animal models of AD [52]. It achieves this by modulating oxidative pathways and preventing neurodegeneration. Additionally, the anti-inflammatory effects of eugenol help suppress microglial activation, which plays a crucial role in the progression of neurodegenerative disorders [53]. Clove is widely used in both culinary and traditional medicinal practices across the world. In cooking, clove is a staple spice in many cuisines, particularly in Asia, the Middle East, and Africa, where it is used to flavor dishes, teas, and desserts [54]. Its aromatic and slightly sweet, yet pungent, taste enhances a variety of foods, including curries, baked goods, and beverages like chai tea. Medically, clove has been an integral part of traditional systems like Ayurveda, Unani, and Chinese medicine. It is used for its antiseptic, analgesic, and digestive properties. Clove oil is applied to relieve toothache and oral infections, while clove teas are consumed to soothe digestive discomfort and boost immunity [55]. In Armenia, clove oil is a key ingredient in an ointment called Yubivaks, which is believed to heal burns. The ointment is currently undergoing preclinical testing for its anti-burn activity [56]. Epidemiological studies on the use of clove and its association with AD incidence in the area where the spice was extensively used are limited but promising. While clinical data in humans are sparse, preliminary findings suggest that clove has potential neuroprotective effects that could lower the risk or progression of AD in populations where clove is a dietary staple or traditional medicine [57]. Furthermore, traditional usage of clove in regions like Southeast Asia, where its consumption is common, aligns with its observed health benefits in experimental studies. Although direct epidemiological evidence linking clove consumption to lower AD incidence is not yet well-established, ongoing pharmacological and biochemical research supports its potential for inclusion in therapeutic strategies against neurodegenerative disease. Owing to its molecular composition, clove contains numerous bioactive compounds with significant neuropharmacological potential (Table 1). These molecules demonstrate antioxidant, anti-inflammatory, and neuroprotective properties. The amounts of molecular components in clove can vary depending on factors, such as the part of the plant used (buds, leaves, or stems) and the method of extraction. For instance, thirty-six constituents were identified from the essential oil of clove buds, and twenty-nine from the essential oil of clove leaves using gas chromatography–mass spectrometry. Major classes of compounds include sesquiterpenes, phenyl propanoids, oxygenated sesquiterpenes, and esters. The composition of major constituents varied between the two oils, with the clove bud essential oil containing eugenol (65.29%), trans-caryophyllene (20.06%), and α-humulene (3.38%), while the clove leaf essential oil contained eugenol (64.47%), trans-caryophyllene (27.19%), and α-humulene (3.62%) [58]. Remarkably, clove components, like eugenol and β-caryophyllene, have been found to be able to cross the blood–brain barrier and have been subjected to studies against glioblastoma [59]. The already mentioned eugenol exhibits neuroprotective, antioxidant, and anti-inflammatory effects by scavenging free radicals, inhibiting neuroinflammation, and modulating calcium channels (Table 1). β-Caryophyllene functions as a CB2 receptor agonist, reducing oxidative stress and neuroinflammation, while gallic acid and quercetin enhance memory and cognitive function by reducing oxidative stress and inhibiting acetylcholinesterase. Luteolin and kaempferol offer neuroprotection through anti-inflammatory pathways, while tannic acid and paeoniflorin mitigate oxidative stress and stabilize cell membranes. Compounds such as isorhamnetin, ellagic acid, and rhamnocitrin demonstrate free radical scavenging and anti-inflammatory properties. Eugenin, oleanolic acid, and asiatic acid contribute to antioxidant defenses and modulate inflammatory pathways, protecting against neurodegenerative diseases. Arjunolic acid also protects against oxidative stress. Together, these phytocompounds highlight clove’s potential as a neuroprotective agent.

**Figure 1:**
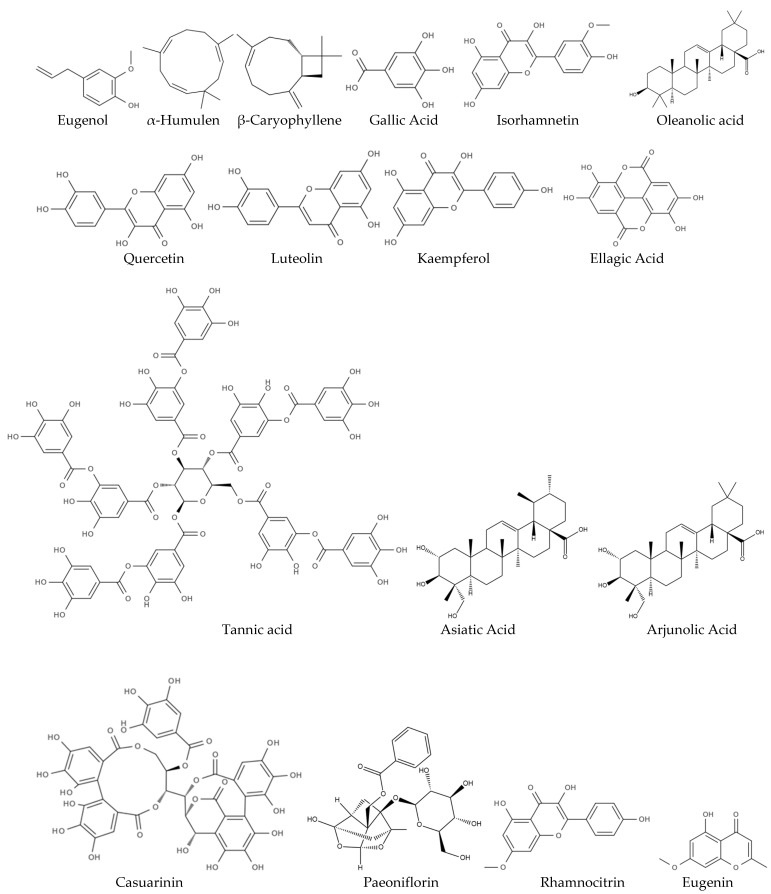
Chemical representations of the phytocompounds found in clove discussed in this section for their neuroprotective properties.

Overall, clove contains a range of bioactive compounds with significant neuropharmacological potential and structural diversity (Figure 1).

## 2. Cloves and Mechanisms of Neuroprotection

### 2.1. Clove Antioxidant Effects

Alzheimer’s disease is a neurodegenerative disease that causes a gradual loss of normal motor and cognitive function. The complex AD pathophysiology involves various factors such as oxidative stress, neuroinflammation, Aβ aggregation, disturbed neurotransmission, and apoptosis. and are not able to cover different aspects of the disease [93]. Clove extracts were evaluated for their effects on hydrogen peroxide-induced oxidative stress in human neuroblastoma SH-SY5Y cell lines, which served as the experimental model. The results demonstrated that both the extracts and key bioactive compounds of *Syzygium aromaticum* effectively reduced ROS, restored mitochondrial membrane potential (MMP), and provided neuroprotection against H_2_O_2_-induced oxidative damage. This protective effect was attributed to the antioxidant properties of the extracts. Additionally, clove extracts significantly diminished lipid peroxidation and restored glutathione levels. The extracts also exhibited anti-acetylcholinesterase activity, anti-glycation effects, and the ability to inhibit Aβ aggregation and fibril formation. The multifaceted neuroprotective mechanisms of clove suggested its potential as a promising candidate for drug development in Alzheimer’s disease [94]. Other studies have indicated that *Syzygium aromaticum* oil may minimize the neurotoxicity caused by acrylamide by reducing oxidative brain damage [95]. As previously mentioned in this work, clove contains bioactive compounds such as phenolic acids, flavonoids, and volatile oils, including eugenol, which demonstrate potent antioxidant properties. These antioxidants play a pivotal role in neuroprotection by scavenging free radicals and mitigating oxidative stress-induced damage in neuronal cells, a key factor in the pathogenesis of AD [96]. Several studies have reinforced the notion that clove extracts exhibit significant antioxidant activity, which may offer neuroprotection by mitigating oxidative damage in the brain, thereby reducing the risk of neurodegenerative diseases [97,98]. Eugenol has been reported to alleviate neuropathic pain [99] and demonstrate anti-amnestic activity in animal models of Alzheimer’s disease, potentially through its antioxidant mechanism [100]. An interesting study demonstrated that chronic administration of clove essential oil improved memory and learning in rats, suggesting its potential role in cognitive enhancement [101]. Another study demonstrated the safety and antidepressant-like effects of *Syzygium aromaticum* essential oil after both acute and long-term treatment. Pronounced antidepressant effects were observed when the oil was administered intragastrically at a dose of 200 mg/kg. The toxicological profile was evaluated through prolonged administration at doses of 100, 200, and 400 mg/kg. Notably, only the highest dose (400 mg/kg) resulted in a significant reduction in body weight, while no significant changes were detected in organ weight ratios or cellular-level markers at any dose. These findings suggest that clove essential oil is a highly effective antidepressant with low toxicity when administered intragastrically [102]. As previously discussed, oxidative stress plays a central role in the pathogenesis of Alzheimer’s disease, contributing to cellular damage through the accumulation of reactive oxygen species. Aging results from the accumulation of damage to cellular proteins and membranes, with ROS-induced oxidative stress being a significant factor in geriatric syndromes and various neurodegenerative diseases. In this context, Sirtuin 1 (SIRT1), an NAD^+^-dependent deacetylase, emerged as a critical mediator in mitigating oxidative damage. SIRT1 is a pivotal regulator of cellular functions associated with aging and neurodegenerative disorders, influencing key signaling pathways related to autophagy, oxidative stress response, and mitochondrial activity—processes central to the development and progression of neurodegenerative diseases, like AD [103]. Expanding on this understanding, Shekhar et al. explored the neuroprotective properties of clove in addressing Aβ_25_-_35_-induced neurotoxicity in neuronal cells. Their key findings confirmed that *Syzygium aromaticum* demonstrates substantial antioxidative capacity, as indicated by its ability to scavenge ROS and enhance the activity of key antioxidant enzymes, including superoxide dismutase, catalase, and glutathione peroxidase. Furthermore, the compound was found to upregulate both recombinant and endogenous SIRT1 activity while simultaneously downregulating γ-secretase, a protein complex involved in amyloid plaque formation. This activation of SIRT1 and reduction in γ-secretase suggested a holistic approach for addressing neurodegenerative diseases with *Syzygium aromaticum*. Clove inhibited the fibrillation and oligomerization of Aβ with high efficacy, and exhibited significant antioxidant activity to protect nerve cells. These findings highlighted its potential use in the treatment of neurodegenerative diseases, particularly AD. In fact, clove, as an ayurvedic product, promises healthy aging with minimal side effects and cost-effectiveness, offering a potential solution to current unmet medical needs [100]. Supporting these observations, other studies have further emphasized the multifaceted neuroprotective properties of *Syzygium aromaticum*. For example, research has shown that clove extract possesses anti-acetylcholinesterase activity, anti-glycation potential, and inhibits amyloid-beta aggregation, all of which contribute to its potential therapeutic benefits in Alzheimer’s disease [104].

Additionally, the activation of SIRT1 by *Syzygium aromaticum* is consistent with findings that SIRT1 plays a crucial protective role against neurodegeneration, enhancing mitochondrial function and reducing oxidative stress [105]. Another study demonstrated that the compounds in *Syzygium aromaticum* effectively inhibited both acetylcholinesterase and butyrylcholinesterase, with stronger inhibition observed for the former enzyme. This suggests the potential of clove oils as an early therapeutic approach for brain dysfunction, particularly in neurodegenerative conditions such as Alzheimer’s disease [106]. A gas chromatography–mass spectrometry analysis identified 58 volatile compounds in clove essential oil. To investigate its antioxidant and anti-aging effects, researchers employed the nematode *Caenorhabditis elegans* as a model organism. Chronic treatment with clove essential oil significantly extended the lifespan and improved the reproductive health of these nematodes. The oil demonstrated antioxidant activity by reducing levels of ROS and by upregulating key antioxidant enzymes, including superoxide dismutase 3 and glutathione S-transferase 4. Additionally, clove essential oil induced the translocation of the DAF-16/FOXO transcription factor from the cytoplasm to the nucleus. DAF-16, the *Caenorhabditis elegans* homolog of the FOXO transcription factor, plays a central role in the insulin/insulin-like growth factor 1 signaling pathway, which regulates longevity, stress resistance, and metabolism. Upon activation, DAF-16 moves into the nucleus and binds specific DNA sequences to promote the expression of genes involved in stress response and lifespan extension [107]. The treatment with clove essential oil leads to germ cell apoptosis in an *acep-1*- and *daf-16*-dependent manner, underscoring the intricate regulatory mechanisms that govern cell death in Caenorhabditis elegans. Although the precise role of *acep-*1 in this context requires further elucidation, the involvement of DAF-16 suggested that clove essential oil may modulate apoptotic pathways through its influence on this transcription factor. Overall, these findings indicate that clove essential oil possesses antioxidant and anti-aging properties, with DAF-16 playing a central role in mediating these effects [108]. The study demonstrated that combining endurance training with clove oil supplementation improved spatial memory in a rat model of Alzheimer’s disease. This combined intervention increased the expression of the α7 nicotinic acetylcholine receptor in the hippocampus—an important receptor involved in cognitive function—while reducing levels of NLRP1 (NOD-like receptor protein 1), a key component of the inflammasome that mediates inflammatory responses. Additionally, the number of dark cells, which indicate cellular damage, decreased. These molecular and cellular changes are likely to contribute to enhanced spatial learning and memory [109].

### 2.2. Clove Anti-Inflammatory Effects

Inflammation plays a critical role in Alzheimer’s disease, contributing significantly to the progression of the condition. Alzheimer’s disease, like other proteinopathic neurodegenerative disorders, is characterized by the accumulation of amyloidogenic proteins. A neuroinflammatory component in Alzheimer’s disease has been known for more than a decade, and although inflammation’s contribution to the disease was initially underappreciated, recent genetic, bioinformatic, and preclinical data now confirm its importance in exacerbating the pathology of the disease. Neuroinflammation in Alzheimer’s disease is primarily driven by the brain’s intrinsic myeloid cells, known as microglia, and this inflammation intensifies as the disease progresses [110]. The effectiveness of some antidementia drugs in animal models of Alzheimer’s disease has been linked to their anti-inflammatory properties. One such example is ellagic acid, a compound found in clove, which has demonstrated an ability to mitigate Alzheimer’s-like behavior in 5xFAD mice. This antidementia effect is attributed to ellagic acid’s ability to reduce inflammatory responses in the brain, decrease oxidative stress, lower amyloid beta deposition, reduce apoptosis, and promote neurogenesis, all of which contribute to the compound’s potential as a therapeutic agent in Alzheimer’s disease [111]. In the context of Alzheimer’s disease, the anti-inflammatory effects of various molecular components found in clove further highlight the potential therapeutic benefits of this natural substance. As mentioned, ellagic acid has shown promise in mitigating Alzheimer’s-like behavior in animal models by reducing inflammation, oxidative stress, and amyloid beta deposition. This anti-inflammatory property is not unique to ellagic acid, as clove is also known for its other molecular components that contribute to such effects. For example, clove essential oil, with its main component, eugenol, is renowned for its analgesic and anti-inflammatory properties. Eugenol has demonstrated significant anti-inflammatory effects [56], which may play a role in protecting the brain from the inflammatory processes that exacerbate Alzheimer’s disease. *Helicobacter pylori* has been associated with an increased risk for various neurological diseases, including Alzheimer’s disease, as well as other conditions like Parkinson’s and multiple sclerosis. This link is primarily through mechanisms of chronic inflammation, systemic inflammation, and neuroinflammation. Given its role in promoting these inflammatory pathways, *Helicobacter pylori* is recognized as a contributing factor to the pathogenesis of neurological disorders. A growing body of evidence suggests that *Helicobacter pylori* infection plays a significant role in the development and progression of Alzheimer’s disease through its impact on chronic inflammation and neuroinflammation. In particular, the presence of *H. pylori* has been associated with increased levels of specific anti-H. pylori antibodies in the cerebrospinal fluid and serum of Alzheimer’s patients, which correlates with disease severity. Studies have also revealed that individuals carrying the ApoE4 polymorphism, the strongest genetic risk factor for Alzheimer’s, are more susceptible to *H. pylori* infection, suggesting that this genetic variant may facilitate the entry of *H. pylori* into the brain. Furthermore, *H. pylori* infection induces systemic inflammation by releasing pro-inflammatory cytokines and toxins, which can cross the blood–brain barrier and disrupt its integrity. This breakdown of the blood–brain barrier, combined with the inflammatory responses triggered by the infection, likely contributes to the neurodegenerative processes observed in Alzheimer’s disease. Thus, eradicating *Helicobacter pylori* can decrease systemic inflammation and improve endothelial function, potentially lowering the risk and severity of these conditions [112]. In this context, a report on the anti-inflammatory activity of clove essential oil in a *Helicobacter pylori* model fits seamlessly within the broader discussion of clove’s potential in combating Alzheimer’s disease [113] studied the anti-inflammatory effects of eugenol clove essential oil, specifically against *Helicobacter pylori*. Their results demonstrated that the essential oil inhibited human erythrocyte hemolysis at concentrations of 4, 8, 16, and 32 μg/L, with inhibition rates ranging from 53.04% to 63.64% (Table 2). Interestingly, sodium diclofenac, a well-established anti-inflammatory drug, showed similar inhibition rates; [114] evaluated the anti-inflammatory effect of nanoemulsion-based gels containing clove and cinnamon essential oils. Even if moderately, a nanogel based on clove showed activity in reducing paw edema, which is a model for inflammation; [115] found that clove essential oil reduced paw swelling by 40–60% in rats, highlighting its anti-inflammatory potential. The gel demonstrated comparable effectiveness to both diclofenac and neomycin, emphasizing the role of clove essential oil in inflammation reduction.

### 2.3. Neurotrophic and Neuropharmacological Effects

Some studies have highlighted the potential of clove extracts in promoting nerve cell growth and survival. Components within clove, particularly eugenol, have shown the ability to induce neurotrophic factors such as brain-derived neurotrophic factor (BDNF) and nerve growth factor (NGF). These neurotrophic factors support neuronal health, aiding in neurogenesis and neuronal regeneration [116,117]. Clove extracts have been suggested to modulate neurotransmitter levels in the brain. In particular, eugenol may influence neurotransmitter systems, including acetylcholine and dopamine pathways, potentially impacting cognitive function and mood regulation. This modulation could contribute to neuroprotective effects [118,119,120]. Several preclinical studies have explored the neuropharmacological effects of clove extracts in animal models. These investigations have reported improvements in cognitive function, memory enhancement, and neurobehavioral outcomes upon administration of clove extract or its constituents. Such effects imply a potential for clove-based interventions in neurodegenerative conditions [121,122,123]. Eugenol has been shown to protect against neuronal death in PC12 cells treated with Aβ. It also mitigated the transcriptional upregulation of the pro-apoptotic protein Bax and the downregulation of the anti-apoptotic protein B-cell lymphoma (Bcl), which are typically induced by Aβ in these cells [124]. Furthermore, eugenol has demonstrated its potential therapeutic effects by improving memory impairment and reducing the number of amyloid plaques, indicating its potential to influence and alter the underlying processes of Alzheimer’s disease [125]. However, the exact mechanisms by which eugenol exerts these effects remain unclear. To further explore these mechanisms, Jung et al. investigated the impact of eugenol on Alzheimer’s disease pathologies and its therapeutic action using a 5×FAD mouse model. Eugenol was found to improve cognitive function, reduce necroptotic cell death, and decrease Aβ accumulation in the 5×FAD mouse model. The therapeutic effects of eugenol may vary depending on the specific brain region targeted. In particular, eugenol exhibited anti-inflammatory properties in the cortex and facilitated microglia-mediated phagocytosis of Aβ in the hippocampus. These findings suggest that eugenol could offer therapeutic benefits for Alzheimer’s disease by modulating the inflammatory response and addressing amyloid-related pathologies [126]. Overall, the effects reported to date in the scientific literature of the different clove extracts and oils, which are due to their molecular components, in in vitro and in vivo models are summarized in the following Table 3.

## 3. Amino Acid and Peptide Components in Cloves with Neuroprotective Potential

Clove is highly valued for its diverse bioactive compounds, including those found in clove essential oil, flavonoids, and phenolic compounds. While plant oils and extracts are commonly used in neuropharmacological studies, the consumption of whole plant material in the case of clove is believed to be linked to neuroprotective effects. In this context, recent studies have highlighted the significant amino acid content across various parts of the plant, such as the buds, fruits, branches, and leaves, which contribute to its nutritional and medicinal properties as summarized in Table 4. Remarkably, amino acids play a crucial role in metabolic processes, immune function, and antioxidant activity, making clove an essential resource for health and wellness applications.

As observed from the data in Table 4, clove is a significant source of both essential and non-essential amino acids, which contribute to its remarkable biological activities. The plant’s various parts, including the buds, fruits, branches, and leaves, contain an array of amino acids that play key roles in human health. Amino acids in clove contribute to energy metabolism, immune system support, tissue repair, neurotransmission, and antioxidant properties, making it a valuable resource for nutritional and pharmacological applications. The buds and fruits of *Syzygium aromaticum* exhibit similar levels of essential amino acids, with total contents of 433.1 and 406.2 mg/kg, respectively. These values are higher than those found in the branches (113.9 mg/kg) and leaves (229.8 mg/kg). The essential amino acids found in clove are of particular interest, as they cannot be synthesized by the human body and must be obtained through diet. These essential amino acids, such as histidine, threonine, valine, and methionine, are critical for various physiological functions including immune modulation, muscle growth, collagen production, and neurotransmitter synthesis. Their presence in clove enhances its potential as a health-promoting ingredient in functional foods and medicinal products. On the other hand, non-essential amino acids like glutamic acid, proline, and alanine also contribute to the plant’s bioactivity. For instance, glutamic acid, a key neurotransmitter, plays an important role in brain function [154]. Similarly, proline supports collagen synthesis, contributing to tissue repair and skin health. These amino acids, though not required through the diet, provide significant benefits to the body, especially in terms of maintaining homeostasis and overall well-being. The presence of these amino acids underscores clove’s therapeutic potential in managing a variety of conditions, from wound healing to inflammation and oxidative stress reduction. The amino acid profile of clove also highlights its versatility as a natural source of bioactive compounds. In addition to these amino acids, clove contains numerous other secondary metabolites, such as flavonoids, phenolic compounds, and essential oils, which further enhance its biological activities, as mentioned in the previous sections of this work. These properties collectively contribute to clove’s reputation as a multifunctional medicinal plant, ideal for both preventive health and therapeutic purposes, including neuroprotection. It is clear that the inclusion of clove in various health and wellness products, particularly in nutritional supplements, could significantly contribute to improving human health.

**Figure 2:**
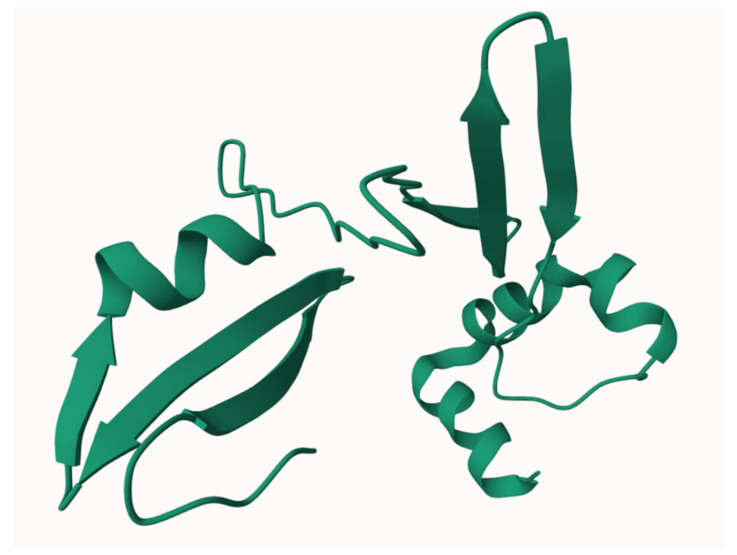
Three-dimensional structure of human ghrelin within its precursor protein, as accessed from https://www.modelarchive.org/doi/10.5452/ma-cfzyt (accessed on 15 February 2025) [156].

The peptide composition of clove is also noteworthy, particularly the identification of ghrelin, an endogenous ligand of the growth hormone secretagogue receptor (Figure 2). In clove, ghrelin was found to have concentrations of 4070.75 ± 664.67 pg/mg in the flower bud [155].

Known for its roles in regulating food intake, energy homeostasis, and insulin release, ghrelin has recently drawn attention for its potential therapeutic effects in neurological disorders, particularly AD. In AD, ghrelin or its receptor agonists have shown promise in attenuating pathology related to amyloid-beta accumulation, tau hyperphosphorylation, neuroinflammation, and cognitive decline [157].

## 4. Conclusions

Clove has been traditionally valued not only for its culinary uses, but also for its medicinal properties. Recent studies have drawn attention to its potential role in managing neurodegenerative diseases like Alzheimer’s disease, with evidence suggesting that its bioactive compounds, particularly eugenol, may offer neuroprotective effects. Although epidemiological studies directly linking clove consumption to AD prevention are still limited, preclinical research demonstrates significant promise. Clove contains a range of bioactive compounds, including flavonoids, phenolic compounds, and amino acids, all of which contribute to its health benefits. Clove is a rich source of both essential and non-essential amino acids, which support numerous biological activities such as energy metabolism, neurotransmission, immune function, and antioxidant action. These amino acids play a crucial role in maintaining metabolic processes and immune function, which are essential for brain health. The amino acid profile and the presence of peptides, like ghrelin, in clove support the role of this spice in brain health and cognitive function. Clove also contains significant levels of eugenol, which is the main compound responsible for its neuroprotective properties. Eugenol has demonstrated antioxidant, anti-inflammatory, and neuroprotective effects in several studies. Eugenol’s ability to modulate inflammation and oxidative stress makes it a promising candidate for AD therapy. Overall, clove’s amino acid content enhances its versatility in supporting cognitive health, alongside its other bioactive compounds, such as flavonoids and phenolic compounds, all of which work synergistically to improve cognitive function, support antioxidant defenses, and reduce neuroinflammation. The presence of essential amino acids, particularly in the buds and fruits of clove, make it a valuable ingredient for both traditional and modern medicinal applications. The combination of antioxidant and anti-inflammatory properties, along with the amino acids’ role in neurotransmission and tissue repair, position clove as a potentially valuable adjunct to AD management, while some of clove’s molecular constituents can contribute to managing other conditions related to oxidative stress, such as cardiovascular disease, by supporting vascular health and reducing inflammation. In conclusion, eugenol and other clove phytocompounds, along with the peptide ghrelin and the specific amino acid composition of clove—particularly its high levels of neuroprotective compounds like glutamic acid—enhance its potential as a therapeutic agent for Alzheimer’s disease. Further clinical research is necessary to fully understand the therapeutic potential of clove, particularly in combination with other neuroprotective agents, and to optimize its use for neurodegenerative diseases. With its diverse bioactive profile, clove holds significant promise as a natural remedy in managing cognitive decline and other neurodegenerative conditions.

## Figures and Tables

**Table 1:** Key molecular components of clove, their potential neuropharmacological effects, mechanisms of action, and supporting references.

Molecular Component	Potential Neuropharmacological Effects	Mechanism of Action	Reference
Eugenol	Neuroprotective, antioxidant, anti-inflammatory	Scavenges free radicals, inhibits neuroinflammation, modulates calcium channels	[60,61,62]
α-Humulen	Antioxidant, anti-inflammatory	NF-κB inhibition, ROS neutralization, COX-2 suppression, membrane disruption.	[55,63,64,65]
β-Caryophyllene	Neuroprotective, anti-inflammatory, anti-anxiety	CB2 receptor agonist, modulates neuroinflammation, reduces oxidative stress	[66,67,68]
Gallic Acid	Antioxidant, anti-apoptotic, memory enhancer	Reduces oxidative stress, prevents neuronal apoptosis	[69,70,71]
Quercetin	Neuroprotective, cognitive enhancer, anti-neuroinflammatory	Inhibits acetylcholinesterase, reduces pro-inflammatory mediators	[66,72,73,74]
Luteolin	Neuroprotective	Inhibits inflammation, promotes neuroprotection, and reduces oxidative stress	[75,76,77]
Tannic acid	Antioxidant, anti-inflammatory, anti-neuroinflammatory	Free radical scavenging, metal chelation, lipid protection, NF-κB inhibition, cytokine reduction, modulates cytokines, inhibits microglial activation	[61,78,79]
Casuarinin	Antioxidant	Scavenges free radicals, reducing oxidative stress	[80]
Paeoniflorin	Antioxidant, anti-inflammatory, neuroprotective	Inhibits pro-inflammatory cytokines, reduces ROS, stabilizes cell membranes	[81]
Kaempferol	Neuroprotective, anti-inflammatory	Suppresses pro-inflammatory pathways, protects against neuronal degeneration	[69,82]
Ellagic Acid	Antioxidant, neuroprotective	Scavenges free radicals, inhibits inflammation, regulates cell cycle	[69,83]
Rhamnocitrin	Antioxidant, neuroprotective	Free radical scavenging, reduction in neuroinflammation	[69,84,85]
Isorhamnetin	Antioxidant, anti-inflammatory	Free radical scavenging, inhibition of pro-inflammatory cytokines	[86,87]
Eugenin	Anti-inflammatory, antioxidant, neuroprotective	Neutralizes reactive oxygen species and reactive nitrogen species (RNS), inhibits the production of pro-inflammatory mediators, interferes with neuroinflammatory pathways	[87,88,89,90]
Oleanolic Acid	Antioxidant, anti-inflammatory	Scavenges free radicals and boosts cellular antioxidant defenses, inhibits the NF-κB pathway and reduces pro-inflammatory cytokines, modulates oxidative stress and inflammation	[87,91,92]
Asiatic Acid	Neuroprotective, anti-inflammatory	Protects neurons from oxidative stress and apoptosis, potentially benefiting neurodegenerative diseases like Alzheimer’s and Parkinson’s, suppresses pro-inflammatory mediators like IL-6 and TNF-α.	[87,91,92]
Arjunolic Acid	antioxidant, anti-inflammatory	Reduces oxidative stress, chelates metal ions and scavenges reactive oxygen species, reduces inflammation in various disease models.	[87,91,92]

**Table 2:** Anti-inflammatory effects of clove essential oil with potential neuroprotective properties.

Model	Effect	Rate	Reference
In vitro (human erythrocyte)	Inhibited human erythrocyte hemolysis	Inhibition by 53.04–63.64%	[113]
In vivo (rat model)	Reduced paw swelling	Reduction by 40–60%	[115]

**Table 3:** Clove extract and oil effects in in vitro and in vivo models.

In Vitro/In Vivo Model	Biological Activity	Plant Part	Extract/Oil	Reference
Antioxidant tests (DPPH, FRAP) (in vitro)	Antioxidant	Buds	Supercritical extract	[94]
Antioxidant analysis (ABTS, DPPH) (in vitro)	Antioxidant	Buds	Essential oil	[95]
Neuron culture, Aβ-induced damage (in vitro)	Neuroprotection (Alzheimer’s disease)	Buds	Ethanol extract	[97]
Primary neuronal cells, scopolamine-induced memory impairment model (in vitro)	Memory enhancement, neuroprotection	Buds	Oil	[98]
Neuronal cell line PC12, stress-induced damage (in vitro)	Neurogenesis, memory improvement	Buds	Eugenol (oil component)	[118]
Neuropathic pain model, eugenol injection in cerebrospinal fluid (in vivo; **rats**)	Pain relief in neuropathic pain	Buds	Eugenol	[96]
Alzheimer’s disease model, Aβ-induced memory impairment (in vivo; **mice**)	Neuroprotection (SIRT1 pathway)	Buds	Extract	[101]
Acrylamide-induced neurotoxicity model (in vivo; **rats**)	Neuroprotection in toxic brain damage	Buds	Oil	[92]
Alzheimer’s disease model, effect of physical exercise (in vivo; **rats**)	Memory enhancement, reduction in damaged cells	Buds	Oil	[106]
Alzheimer’s disease model, mitochondrial function analysis (in vivo; **rats**)	Memory restoration, apoptosis reduction, improved mitochondria	Buds	Extract	[49]

**Table 4:** Amino acid composition of clove in its different parts like buds, fruits, and branches [127,128], as well as their biological activities connected to their neuroprotective role [87].

Amino Acid	Buds (mg/kg)	Buds (mg/kg)	Fruits (mg/kg)	Branches (mg/kg)	Leaves (mg/kg)	Biological Properties	Reference
**Aspartic Acid**	111.6	42.8	105.4	-	-	Supports metabolism and neurotransmission	[129]
**Serine**	69.8	80.5	41.5	57.9	37.9	Supports protein synthesis and acts as a precursor for neurotransmitters	[130]
**Glutamic Acid**	93.8	91.3	74.1	64.2	66.4	Functions as an excitatory neurotransmitter and antioxidant	[131]
**Glycine**	61.2	-	42.3	40.5	41.4	Neurotransmitter, anti-inflammatory, cytoprotective, immunomodulatory, metabolic precursor	[132,133,134]
**Histidine**	121.6	-	118.8	121.2	120.6	Encompasses neurotransmitter synthesis, enzymatic catalysis, metal ion chelation, and plays a role in the modulation of immune responses and growth	[135,136]
**Arginine**	133.1	113.7	96.1	250.1	89.9	Encompasses nitric oxide production, immune enhancement, antimicrobial action, and metabolic regulation	[137,138]
Threonine *	38.4	260.4	40.1	-	34.8	Plays a critical role in protein synthesis, immune function, and various metabolic pathways	[139]
**Alanine**	94.5	-	93.8	52.3	55.2	Supports gluconeogenesis, insulin secretion, immune function, and longevity	[140]
**Tyrosine**	77.5	40.0	69.3	64.1	66.7	Precursor for hormones like dopamine and adrenaline. Affects cognition, thermoregulation, neurotransmission, and may influence lifespan at varying doses	[141]
Valine *	65.9	106.1	50.2	45.7	44.9	Contributes to muscle growth, tissue repair, has antioxidant properties, and activates NRF2 to improve cellular health and growth	[142,143]
Methionine *	63.3	14.1	62.8	-	-	Acts as an antioxidant and supports liver detoxification	[144,145]
Lysine *	68.9	-	68.5	68.2	66.8	Essential for protein synthesis, longevity, metabolism, and tissue repair; plays a significant role in antioxidant and anti-inflammatory activities	[146,147]
Isoleucine *	59.8	16.8	53.1	-	-	Branched-chain amino acid, affects glucose metabolism, insulin resistance, and may play a role in aging	[143,148]
Leucine *	61.8	27.7	56.8	-	-	Influences lifespan, metabolism, muscle function, and longevity regulation pathways	[143,149]
Phenylalanine *	75.0	21.1	74.8	-	83.3	Precursor for neurotransmitters like dopamine and norepinephrine, antioxidant.	[150]
**Proline**	154.9	-	203.7	97.6	63.7	Enhances collagen synthesis and cellular repair, supports antioxidant activity, and contributes to metabolic regulation.	[151]
Tryptophan *	-	12.1	-	-	-	Recursor to melatonin, serotonin, and vitamin B3. It influences aging, neurotransmitter synthesis, mood regulation, and sleep cycles.	[152,153]
**Total Amino Acids (TAA)**	1351.1	830.2	1251.2	861.8	771.6		
**Essential Amino Acids (EAA)**	433.1	461.9	406.9	113.9	229.8		

* The asterisk denotes essential amino acids.

## Data Availability

Not applicable.
